# Phenotypic and functional analysis of *SHANK3* stop mutations identified in individuals with ASD and/or ID

**DOI:** 10.1186/s13229-015-0020-5

**Published:** 2015-04-29

**Authors:** Daniela M Cochoy, Alexander Kolevzon, Yuji Kajiwara, Michael Schoen, Maria Pascual-Lucas, Stacey Lurie, Joseph D Buxbaum, Tobias M Boeckers, Michael J Schmeisser

**Affiliations:** Institute for Anatomy and Cell Biology, Ulm University, Albert-Einstein-Allee 11, D-89081 Ulm, Germany; Seaver Autism Center for Research and Treatment, Icahn School of Medicine at Mount Sinai, One Gustave L. Levy Place, New York, NY 10029 USA; Friedman Brain Institute, Icahn School of Medicine at Mount Sinai, One Gustave L. Levy Place, New York, NY 10029 USA; Mindich Child Health and Development Institute, Icahn School of Medicine at Mount Sinai, One Gustave L. Levy Place, New York, NY 10029 USA; Department of Psychiatry, Icahn School of Medicine at Mount Sinai, One Gustave L. Levy Place, New York, NY 10029 USA; Department of Pediatrics, Icahn School of Medicine at Mount Sinai, One Gustave L. Levy Place, New York, NY 10029 USA; Neuroscience Division, Center for Applied Medical Research, CIMA, University of Navarra, Av. Pio XII 55, 31008 Pamplona, Spain; Department of Neuroscience, Icahn School of Medicine at Mount Sinai, One Gustave L. Levy Place, New York, NY 10029 USA; Department of Genetics and Genomic Sciences, Icahn School of Medicine at Mount Sinai, One Gustave L. Levy Place, New York, NY 10029 USA

**Keywords:** ASD, Autism, SHANK3, Intellectual disability, Nucleus, Dendrite, Spine, Synapse

## Abstract

**Background:**

SHANK proteins are crucial for the formation and plasticity of excitatory synapses. Although mutations in all three *SHANK* genes are associated with autism spectrum disorder (ASD), *SHANK3* appears to be the major ASD gene with a prevalence of approximately 0.5% for *SHANK3* mutations in ASD, with higher rates in individuals with ASD and intellectual disability (ID). Interestingly, the most relevant mutations are typically *de novo* and often are frameshift or nonsense mutations resulting in a premature stop and a truncation of SHANK3 protein.

**Methods:**

We analyzed three different *SHANK3* stop mutations that we identified in individuals with ASD and/or ID, one novel (c.5008A > T) and two that we recently described (c.1527G > A, c.2497delG). The mutations were inserted into the human *SHANK3a* sequence and analyzed for effects on subcellular localization and neuronal morphology when overexpressed in rat primary hippocampal neurons.

**Results:**

Clinically, all three individuals harboring these mutations had global developmental delays and ID. In our *in vitro* assay, c.1527G > A and c.2497delG both result in proteins that lack most of the SHANK3a C-terminus and accumulate in the nucleus of transfected cells. Cells expressing these mutants exhibit converging morphological phenotypes including reduced complexity of the dendritic tree, less spines, and less excitatory, but not inhibitory synapses. In contrast, the truncated protein based on c.5008A > T, which lacks only a short part of the sterile alpha motif (SAM) domain in the very SHANK3a C-terminus, does not accumulate in the nucleus and has minor effects on neuronal morphology.

**Conclusions:**

In spite of the prevalence of SHANK3 disruptions in ASD and ID, only a few human mutations have been functionally characterized; here we characterize three additional mutations. Considering the transcriptional and functional complexity of *SHANK3* in healthy neurons, we propose that any heterozygous stop mutation in *SHANK3* will lead to a dysequilibrium of SHANK3 isoform expression and alterations in the stoichiometry of SHANK3 protein complexes, resulting in a distinct perturbation of neuronal morphology. This could explain why the clinical phenotype in all three individuals included in this study remains quite severe - regardless of whether there are disruptions in one or more SHANK3 interaction domains.

**Electronic supplementary material:**

The online version of this article (doi:10.1186/s13229-015-0020-5) contains supplementary material, which is available to authorized users.

## Background

Autism spectrum disorder (ASD) is a neuropsychiatric condition manifesting in early development and is characterized by two core features: A) persistent deficits in social interaction and communication and B) the presence of restricted interests and/or repetitive behaviors [1]. The strong involvement of genetics in the development of ASD is supported by the identification of causative genetic abnormalities in more than 20% of cases, with a significant number of the identified genes encoding proteins required for the correct formation, maturation, and maintenance of synaptic connections in the brain [2-9]. Among these, the *SHANK* gene family plays a decisive role because diverse genetic variation in *SHANK1*, *SHANK2*, and *SHANK3* - all encoding large postsynaptic scaffold proteins - has been identified in individuals with ASD [10-16]. A crucial role of *SHANK3* mutations in this context is supported by the following three facts: 1) *SHANK3* haploinsufficiency is the critical factor for the development of neuropsychiatric symptoms in 22q13 deletion syndrome, also known as Phelan-McDermid syndrome, 2) the current prevalence for *SHANK3* mutations in individuals with ASD in general is between 0.5% and 0.7%, and 3) data indicate that a *SHANK3* mutation is present in approximately 2% of individuals with both ASD and intellectual disability (ID) [16-18].

Some individuals diagnosed with either ASD, ID, or both harbor frameshift or nonsense mutations in *SHANK3* resulting in a premature stop codon and causing a truncation of SHANK3 protein [15-17,19-22]. However, only a few studies have thus far addressed the impact of such mutations and their corresponding truncated proteins on neuronal function and morphology [15,19,22-25]. In this context, the *de novo* exon 21 frameshift mutation c.3679_3680insG - identified in two brothers diagnosed with both ASD and ID [15] - and the *de novo* exon 21 nonsense mutation c.3349C > T - identified in three brothers, all of them diagnosed with ID, with two having an additional diagnosis of schizophrenia (SCZ) [19] - have been most intensely studied up to date [15,19,23-25]. Insertion of either stop mutation into the rat *Shank3a* sequence at the corresponding sites results in the expression of truncated Shank3a variants lacking distinct parts of the C-terminus, a region crucial for appropriate synaptic targeting and assembly [26-29]. In contrast to wild-type Shank3a, Shank3a harboring either c.3679_3680insG or c.3349C > T mutations no more cluster at synapses, but rather distribute in the somatodendritic compartment and localize to the nucleus when overexpressed in primary hippocampal neurons [15,19,23-25]. Overexpression of Shank3a harboring the c.3679_3680insG mutation affects growth cone mobility and negatively interferes with synaptic transmission and transsynaptic signaling; the same mutation leads to reductions in the number of excitatory synapses and dendritic spines [15,23,24]. Shank3a harboring the c.3349C > T mutation impairs the ability of Shank3a to promote the outgrowth of primary neurites, results in a less complex dendritic arbor, and leads to a specific reduction of excitatory, but not inhibitory synapses [19,25]. *In vitro* examination of a *de novo* exon 21 nonsense mutation c.2997C > G identified in a boy with ID demonstrated a reduction in neurite nodes, tips, and length, at early stages of neuronal differentiation [22]. Taken together, these *in vitro* studies show that truncations of the distal C-terminus of Shank3a, caused by the c.3679_3680insG, c.3349C > T or c.2997C > G mutations are sufficient to disrupt neuronal morphology when the truncated variant is overexpressed in primary neuronal cultures.

However, the three stop mutations studied to date all affect exon 21 of *SHANK3*, but less is known about mutations identified in ASD and/or ID that affect other parts of the gene [15-17,19-22]. It is therefore of high interest to evaluate additional mutations, including those that disrupt other exons of *SHANK3* and to identify converging and/or distinct neuronal pathologies.

We inserted three mutations identified in subjects with ASD and/or ID into the human *SHANK3a* sequence: a nonsense mutation affecting exon 12 (c.1527G > A), a frameshift mutation affecting exon 21 (c.2497delG) - both recently described [17] - and one novel nonsense mutation affecting exon 22 (c.5008A > T). Clinical assessment of the corresponding subjects was followed by characterization of the impact of the three truncated SHANK3a variants with respect to subcellular localization, dendritic branching, and spine and synapse formation, when overexpressed in rat primary hippocampal neurons with a wild-type Shank3 background.

## Methods

### Participant information

The Institutional Review Board (IRB) of Icahn School of Medicine at Mount Sinai approved all studies involving humans, and all subjects were recruited under an IRB approved protocol as part of ongoing studies in Phelan-McDermid syndrome at the Seaver Autism Center for Research and Treatment at the Icahn School of Medicine at Mount Sinai with parents providing informed consent. An inter-disciplinary evaluation team conducted comprehensive assessments using the following clinical evaluation tools: (1) *Clinical genetic* evaluations and dysmorphology exams; (2) *Neurological examination* to evaluate gross motor skills and gait, fine motor coordination, cranial nerves, and deep tendon reflexes; (3) *ASD focused diagnostic evaluation* using the Diagnostic and Statistical Manual for Mental Disorders-IV (DSM-IV), the Autism Diagnostic Observation Schedule-2 (ADOS-2), and the Autism Diagnostic Interview Revised (ADI-R); (4) *Cognitive testing* using the Mullen Scales of Early Learning; (5) The *Vineland Adaptive Behavior Scale*s-II, Survey Edition, to evaluate independence in daily life skills, including communication, socialization, and motor skills; (6) *Medical record review* including analyzing any results from electroencephalographic and brain imaging studies; and (7) *Genetic testing* for confirming mutation and *de novo* origin, using Sanger sequencing.

### Vector constructs

Human *SHANK3a* complementary DNA (cDNA) based on NP_277052.1 was re-designed in collaboration with GeneArt® (Life Technologies, Carlsbad, CA, USA) for optimized GC content, and a Myc-tag was added immediately after the initiation codon. Subcloning was performed using In-Fusion HD (Clontech Laboratories, Mountain View, CA, USA). The entire *SHANK3a* cDNA was amplified using the following set of primers: 5′-GTCCGGACTCAGATCTATGGAGCAGAAGCTGATCAG-3′ and 5′-GTCGACTGCAGAATTCTCAGCTGCCGTCCAGCTGT-3′, and further inserted into the pAcGFP1-C1 (Clontech Laboratories, Mountain View, CA, USA) vector using Bgl2 and EcoR1 sites. The sequence was confirmed by Sanger sequencing. The c.1527G > A variant was generated by using the primer set 5′-GCTTCTGaGAGGGCACCGTGAAG-3′ and 5′-TGCCCTCtCAGAAGCCGCCctcg-3′. The c.2497delG variant was generated by inserting a fragment containing the deletion followed by authentic human *SHANK3* cDNA corresponding to the sequence from immediately after the variation to the predicated premature termination codon. The c.5008A > T variant was generated by using the primer set 5′-GTGGTCCtAGTTCGACGTGGGCGACTGG-3′ and 5′-CGAACTaGGACCACAGCTGCAGGGGTTT-3′. The eGFP-Shank3a and DenMark constructs have been described previously [29,30].

### Antibodies

A novel polyclonal antibody directed against the rat Shank3a N-terminus (aa 333-470) was generated for this study according to the antibody production and purification protocol described in [31]. The anti-Shank3 PRC antibody has been described previously [31]. The following primary antibodies were purchased from commercial suppliers: anti-histone H3 (Cell Signaling Technology, Danvers, MA, USA), anti-green fluorescent protein (GFP) (Clontech, Laboratories, Mountain View, CA, USA), anti-c-Myc (Roche Applied Science, Mannheim, Germany), as well as anti-GAPDH, anti-VGLUT1, and anti-VGAT (all from Synaptic Systems, Goettingen, Germany).

### Biochemistry

For whole culture extracts, transfected HEK293T cells were lysed in Triton X-100 Lysis Buffer (150 mM NaCl, 50 mM Tris HCl, 1% Triton X-100, pH 8,0, protease inhibitor mix, Roche Applied Science, Mannheim, Germany). The NE-PER Nuclear and Cytoplasmic Extraction Reagents (Thermo Scientific, Bonn, Germany) were further used to obtain nuclear and cytoplasmic fractions from transfected HEK293T cells. Protein concentrations were determined by Bradford protein assay, and the same amount of protein was loaded per lane for SDS-PAGE. Western blot analysis was conducted following standard protocols. HRP-conjugated secondary antibodies (Dako, Glostrup, Denmark) and the SuperSignal detection system (Thermo Scientific, Bonn, Germany) were used to visualize protein bands on X-ray films (GE Healthcare, Freiburg, Germany).

### Animal experiments

All animal experiments in this study were performed based on the guidelines for the welfare of experimental animals issued by the Federal Government of Germany and by the local ethics committee (Ulm University), ID Number: 0.103.

### Cell culture

HEK293T cells were maintained in DMEM at 37°C in 5% CO_2_. The preparation of hippocampal cultures from rat was performed at embryonic stage 18 (E18) as described previously [32]. In brief, hippocampal neurons were seeded on poly-l-lysine (0.1 mg/ml, Sigma-Aldrich, Steinheim, Germany)-coated glass coverslips. Cells were grown in neurobasal medium, complemented with B27 supplement, 0.5 mM L-glutamine and penicillin/streptomycin at 100 U/ml (all reagents from Life Technologies, Darmstadt, Germany), and maintained at 37°C in 5% CO_2_.

### Immunocytochemistry

Immunocytochemistry was performed as described previously with minor modifications [33]. Cultured cells were fixed with 4% paraformaldehyde (PFA)/1.5% sucrose in phosphate-buffered saline (PBS) at RT for 20 min and processed for immunocytochemistry. After permeabilization of the cells with 0.1% Triton X-100 in PBS for 5 min, blocking was performed using 5% FCS in PBS, followed by the primary antibody at 4°C overnight. Washing with PBS was followed by incubation with the secondary antibody coupled to Alexa Fluor® 488, 568, or 647 (all from Life Technologies, Darmstadt, Germany) for 1 h at room temperature. The actin cytoskeleton was visualized by Alexa Fluor® 647 Phalloidin in some experiments. Cell nuclei were counterstained with 4′,6-diamidino-2-phenylindole (DAPI), and after further washing steps, cells were mounted in mowiol. Images were captured using an upright fluorescence microscope (Axioskop 2, Zeiss, Oberkochen, Germany) and Axiovision software (Zeiss, Oberkochen, Germany).

### Transfections

Vector constructs were transfected into HEK293T cells using PolyFect reagent (Qiagen, Hilden, Germany) as described previously [34] or into hippocampal neurons using Lipofectamine 2000 reagent (Life Technologies, Darmstadt, Germany).

### Analysis of neuronal morphology

All analyses were done in a blinded fashion. Sholl analysis was performed as described previously [35]. Concentric circles (15, 30, 45, 60, 75, 90, 105, 120, 135, and 150 μm in diameter) were drawn around the soma of each neuron included in the analysis. The number of all dendrites crossing each circle was counted manually. For analysis of spines and filopodia, two secondary dendrites were randomly chosen per neuron and dendritic protrusions were counted manually among approximately 35-μm-long segments per dendrite. Dendritic protrusions shorter than 1 μm with clearly visible head and neck were counted as spines, and dendritic protrusions longer than 1 μm and devoid of head and neck were counted as filopodia. For analysis of synaptic contacts, four secondary dendrites were randomly chosen per neuron and signals positive for either VGLUT1 (excitatory contacts) or VGAT (inhibitory contacts) were manually counted among approximately 50-μm-long segments per dendrite.

### Statistical analysis

For all analyses in primary culture, five to eight neurons from three independent experiments were analyzed per condition; ‘*n*’ therefore ranged between 15 and 22. GraphPad Prism 5.01 (GraphPad Software, La Jolla, CA, USA) was used for all statistical analyses. Depending on the datasets, analysis was performed with unpaired Student’s *t*-test or one-way ANOVA with Bonferroni *post hoc* test if data were normally distributed or with the Mann-Whitney *U* test or the Kruskal-Wallis test with Dunn’s multiple comparison *post hoc* test if data were not normally distributed.

## Results

### Clinical phenotype

Participant 1 (P1) (c.1527G > A) is a 5-year-old male that we have previously described [17] whose parents first became concerned about his development due to social and language delays. Prior to identifying a *SHANK3* mutation using whole exome sequencing, a 17q12 microduplication had been detected and previously described [36]. As such, caution is warranted in making direct phenotypic comparisons across the mutations described here and elsewhere. P1 has the use of approximately 5 to 10 words to identify objects when prompted, but he does not have any communicative language. While he is able to initiate interactions and make eye contact at times, he does not engage in reciprocal social interaction and is not interested in other children. Behaviorally, he shows significant repetitive behavior, including forced expirations, pacing, and opening and closing doors. He tends to play in a patterned way and has pronounced deficits in imitation, pretend play, and symbolic play. There is also significant difficulty sustaining focus, and he can be quite hyperactive. P1 met criteria for autistic disorder on the ADI-R, ADOS-G, and DSM-IV (Additional file 1: Table S1). With regard to his adaptive behavior, P1 demonstrates difficulties among all domains and overall functioning is low. He requires substantial support with the majority of self-care tasks, including feeding and dressing, and he is not toilet trained. He does eat and sleep well, however. Cognitively, P1 demonstrates abilities ranging from a 6-month-old (expressive language) to 15-month-old (fine motor) level on the Mullen, with significant variability in his profile. He shows relative strengths in fine and gross motor skills, understands basic instructions, and expresses himself with vocalizations and some gestures. He has difficulty labeling most objects and does not follow two-step instructions. P1 has no chronic medical problems. He has never had a seizure, and an electroencephalography (EEG) in the past was within normal limits. A past magnetic resonance imaging (MRI) revealed diffuse ventricular enlargement and thinning of the parieto-occipital white matter and corpus callosum. P1 has no renal or cardiac abnormalities but is reported to be allergic to penicillin. He has hypotonia and mild nonspecific gait abnormalities with toe walking. On dysmorphology exam, P1 has long eyelashes, protruding ears, broad nasal bridge, full lips, and macrocephaly. Skin exam revealed two café au lait spots on his back (Additional file 2: Table S2).

Participant 2 (P2) (c.2497delG) is a 7-year-old male also previously described by us [17] whose parents first became concerned about his development at 6 months old due to hypotonia and significant difficulty eating solid foods. Although he is nonverbal, he developed some adapted sign language to a maximum of ten adapted signs. His receptive language abilities (age equivalent: 13 months) are stronger than his expressive abilities (age equivalent: 5 months), but he does not follow simple directions (see below). P2 is able to make eye contact and engage with others, and when interested, he will smile and wave. He will repetitively put nonfood objects in his mouth and is also described as being obsessed with electronics and phones, while there are no other compulsive or ritualistic behaviors. Sensory symptoms include a high pain threshold, and he does not like to wear shoes or socks. He also has poor thermoregulation and gets cold very easily. On autism-focused diagnostic instruments, P2 met criteria on all three ADI-R domains (social, communication, and repetitive behaviors), but not on the ADOS-G or the DSM-IV, and overall, he did not meet overall consensus criteria for an autism spectrum disorder (Additional file 1: Table S1). In terms of associated features, P2 has a very limited attention span and is unable to focus for more than 45 to 60 s at a time. He is extremely active and restless and has the potential to be aggressive. Sleeping difficulties are prominent and characterized by early morning wakening. His adaptive functioning is low overall; P2 is unable to dress or feed himself, and he is not toilet trained. His gait is apraxic with left foot dragging and pronation of his feet bilaterally. However, he is able to ride a tricycle and to climb stairs with alternating feet. Cognitive testing shows that P2 is functioning at a 5-month-old (expressive language) to 20-month-old (gross motor) level on the Mullen. He is able to understand some simple commands and questions, in addition to identify common objects. However, he is not able to follow directions or label body parts. In terms of medical features, P2 has been diagnosed with a seizure disorder and has localized sleep-potentiated epileptiform discharges mainly in the midline and central regions during slow wave sleep. There has never been a seizure observed on EEG, although clinically, they are accompanied by myoclonic twitching of his ankle and face. A previous MRI study showed evidence of leukodystrophy but was otherwise within normal limits. There was evidence of a significant regression in sign language and motor skills around 6 years old in the context of an increase of seizure activity. At one point during that period, P2 stopped walking for about 6 weeks and then slowly regained his ambulatory skills as the seizures were better controlled. A full metabolic disease workup was negative, but P2 has a short stature, and a feeding tube is in place. He has gastrointestinal symptoms manifested by periods of fluctuating diarrhea and constipation and severe gastroesophageal reflux disease (GERD) treated with famotidine. He has a history of recurring ear infections requiring myringotomy tube placement at 2 years old. He has no renal or cardiac problems, and no allergies to food or medications. In terms of dysmorphic physical features, P2 has short stature and mild dolicocephaly (Additional file 2: Table S2).

Participant 3 (P3) (c.5008A > T) is a 12-year-old male whose parents first became concerned about his development at 2 ½ years old due to language regression and feeding problems. He had developed approximately 10 to 15 words by 18 months but subsequently lost all expressive languages. His language comprehension is also limited, but he is reportedly able to understand approximately 40 signs. He can express his needs through a Picture Exchange Communication System (PECS), but not consistently. P3 interacts with family members but is generally uninterested in social interaction. He exhibits significant repetitive behaviors and will watch the same portion of a television show or movie repeatedly. He also engages in motor stereotypies, including hand flapping and rocking. He may grind his teeth, chew compulsively, and make repeated stereotypic vocalizations. He is also sensory seeking through tactile modalities, including pressure, and has a high pain threshold. P3 met full criteria for autistic disorder on the ADI-R, ADOS-G, and DSM-IV (Additional file 1: Table S1). In terms of associated features, he may become aggressive and rarely bites others but is not self-injurious. There is no motoric hyperactivity, but he has significant sleep difficulties, including delays in sleep onset and early morning awakening. His adaptive functioning is low in all domains. He continues to have difficulty eating solid food and is unable to manipulate utensils. He has hypotonia but is able to ride a tricycle. P3 is not toilet trained. Cognitive functioning is also low in the 8-month (receptive language) to 30-month (gross motor) range on the Mullen. P3 was diagnosed with epilepsy at 7 years old after a 48-h video EEG showed clinical events but without electrographic correlation. A subsequent MRI that year revealed a venous angioma. During the same period of time, P3 experienced a regression in motor skills where he previously was able to hold a pencil and write his name but then lost this skill. At 8 years old, he experienced a 9-month period of significant muscle weakness and was unable to ambulate for 6 months. He continues to have hypotonia with mild nonspecific gait abnormalities, including intoeing. He has no renal and no cardiac abnormalities. P3 is reportedly allergic to casein and gluten and on a restricted diet. He also has a history of chronic diarrhea (seven to eight times a day) and recurring ear infections, which have both improved. In terms of dysmorphic features, P3 is noted to have deep set eyes, fifth finger clinodactyly, and second toes overlapping (Additional file 2: Table S2).

### Domain composition and biochemical expression analysis of SHANK3 variants

**Figure 1:**
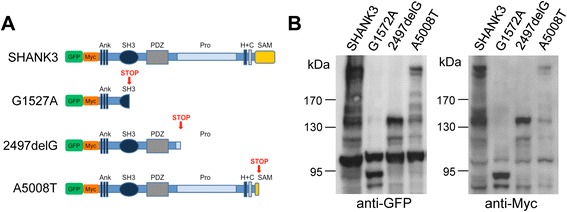
Domain composition and expression analysis of SHANK3 variants. **(A)** Schematic overview of the SHANK3 constructs/fusion proteins used for overexpression experiments in this study. All constructs harbor an N-terminal GFP tag followed by a Myc-tag (indicated in green and orange, respectively). Domains are marked as it follows: Ank, ankyrin repeat domain; SH3, Src homology 3 domain; PDZ, postsynaptic density 95/discs large/zonula occludens-1 domain; Pro, proline-rich region; H, Homer binding site; C, cortactin binding site; and SAM, sterile alpha motif domain. SHANK3 is based on the full-length human *SHANK3a* sequence containing all of these domains. The truncated SHANK3 variants are identified as G1527A, 2497delG, and A5008T, and each is based on the *SHANK3a* sequence harboring either c.1527G > A, c.2497delG, or c.5008A > T, respectively. Predicted premature stop sites are marked by STOP in red. **(B)** Functional validation of the SHANK3 constructs in HEK293T cells via detection of the overexpressed fusion proteins SHANK3, G1527A, 2497delG, and A5008T by Western blot analysis using either anti-GFP (left panel) or anti-Myc (right panel) antibodies. kDa, kilodalton.

The full length human SHANK3a protein contains N-terminal ankyrin repeats (Ank) followed by a Src-homology 3 domain (SH3), a postsynaptic density (PSD)-95/Dlg1/ZO1 domain (PDZ), a proline-rich region (Pro), Homer and cortactin binding sites (H + C) and a C-terminal sterile alpha motif (SAM) domain (Figure 1A). In the aforementioned individuals, the c.1527G > A nonsense mutation affects exon 12 of *SHANK3* resulting in a truncation within the SH3 domain, the c.2497delG frameshift mutation in exon 21 disrupts the proline-rich region, and the c.5008A > T nonsense mutation in exon 22 disrupts the SAM domain. The corresponding fusion proteins, named SHANK3, G1527A, 2497delG, and A5008T in this study, have been engineered to harbor an N-terminal GFP-tag followed by a Myc-tag. Both G1527A and 2497delG are predicted to lack the SHANK3 C-terminus including the proline-rich region, Homer and cortactin binding sites and the C-terminal SAM domain (and the PDZ domain and parts of the SH3 domain in case of G1527A), while A5008T is only predicted to lack most of the SAM domain (Figure 1A). To validate the functionality of our expression constructs, we transfected HEK293T cells and detected the corresponding fusion proteins by Western blot analysis of whole cell extracts using either anti-GFP (Figure 1B, left panel; Additional file 3: Figure S1A), anti-Myc (Figure 1B, right panel), or polyclonal anti-Shank3 antibodies (Additional file 3: Figure S1B,C). All four antibodies detected wild-type SHANK3 at the correct size (predicted molecular weight including tags approximately 220 kD). Both anti-GFP and anti-Myc antibodies also detected all three truncated fusion proteins G1527A (approximately 90 kD), 2497delG (approximately 130 kD), and A5008T (approximately 215 kD) at the correct size (Figure 1B). The anti-Shank3 PRC antibody recognized 2497delG and A5008T but failed to detect G1527A due to the lack of antigen sequence within this fusion protein (Additional file 3: Figure S1B, for antigen sequence, see [31]). We therefore used a novel anti-Shank3 N-term antibody, which successfully detected G1527A (Additional file 3: Figure S1C).

### Nuclear accumulation of truncated SHANK3 variants G1527A and 2497delG

**Figure 2:**
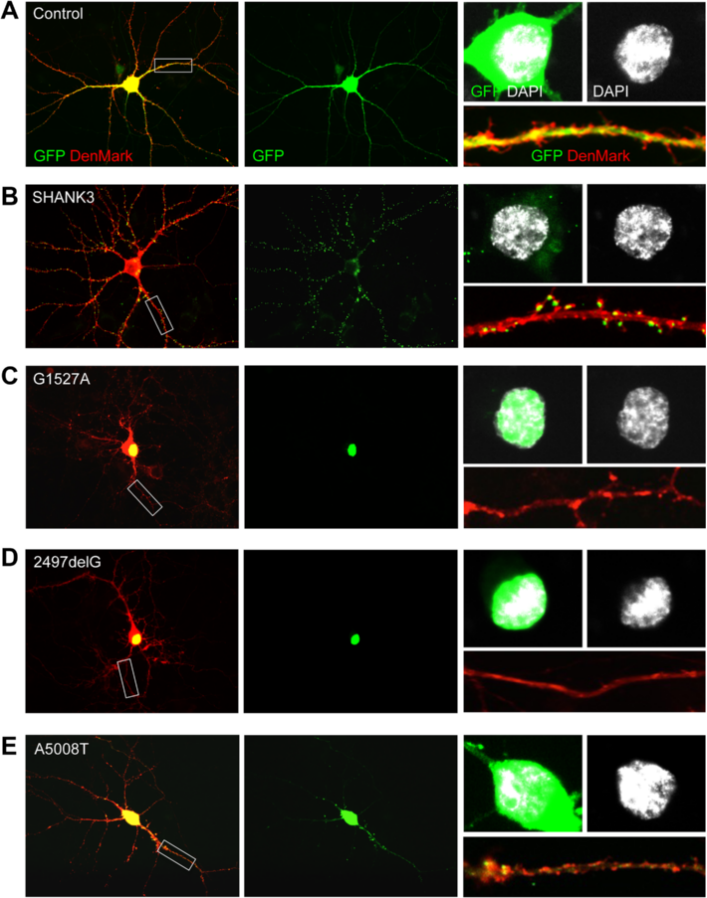
Nuclear accumulation of truncated SHANK3 variants G1527A and 2497delG. **(A-E)** Subcellular distribution of empty vector-based GFP (Control) **(A)**, full-length GFP-Myc-SHANK3 (SHANK3) **(B)**, or the truncated GFP-Myc-SHANK3 variants G1527A **(C)**, 2497delG **(D)**, and A5008T **(E)**, after transient overexpression (DIV11-14) in rat primary hippocampal neurons. All neurons shown were co-transfected with the DenMark construct [30] containing an mCherry sequence to demarcate dendrites and spines in red. They were further immunostained for VGLUT1 (not shown), and nuclei were visualized by DAPI. In each panel **(A-E)**, one representative co-transfected neuron is depicted. The picture on the left is a merge of the GFP and DenMark signals, while the picture in the middle shows only the GFP signal. The upper insets on the right show a merge of GFP and DAPI signals or the DAPI signal alone, and the lower inset on the right shows a representative secondary dendrite as a merge of the GFP and DenMark signals. Note the strong overlap of both G1527A and 2497delG with the DAPI signal.

We transfected rat primary hippocampal neurons at DIV11 and analyzed subcellular localization of SHANK3, G1527A, 2497delG, and A5008T at DIV14. The dendritic tree and dendritic spines were visualized by co-transfection of DenMark [30], with empty vector used as Control (Figure 2A,B,C,D,E). SHANK3 localized to the somatodendritic compartment where it mainly formed cluster-like structures within dendrites, spines, and at excitatory synapses (Figure 2B, Additional file 4: Figure S2A,B) - as previously shown for its homologues in mouse and rat [23,26,37,38]. A5008T also localized to the somatodendritic compartment and appeared in cluster-like structures within dendrites and at excitatory synapses - albeit to a much lesser extent than SHANK3 (Figure 2E, Additional file 4: Figure S2A,B). In contrast, G1527A and 2497delG exclusively co-localized with DAPI-positive nuclei in transfected neurons, thus implicating nuclear or perinuclear enrichment of both fusion proteins (Figure 2C,D). To further investigate this phenomenon, overexpression experiments were performed in HEK293T cells, which better allows biochemical detection of the fusion proteins in subcellular fractions due to higher transfection rates. In line with our observations in primary neuronal cultures, both G1527A and 2497delG exclusively co-localized with DAPI-positive nuclei and were found biochemically enriched within the nuclear fraction (Additional file 5: Figure S3A,B).

### Overexpression of truncated SHANK3 variants results in distinct alterations of dendritic tree complexity

**Figure 3:**
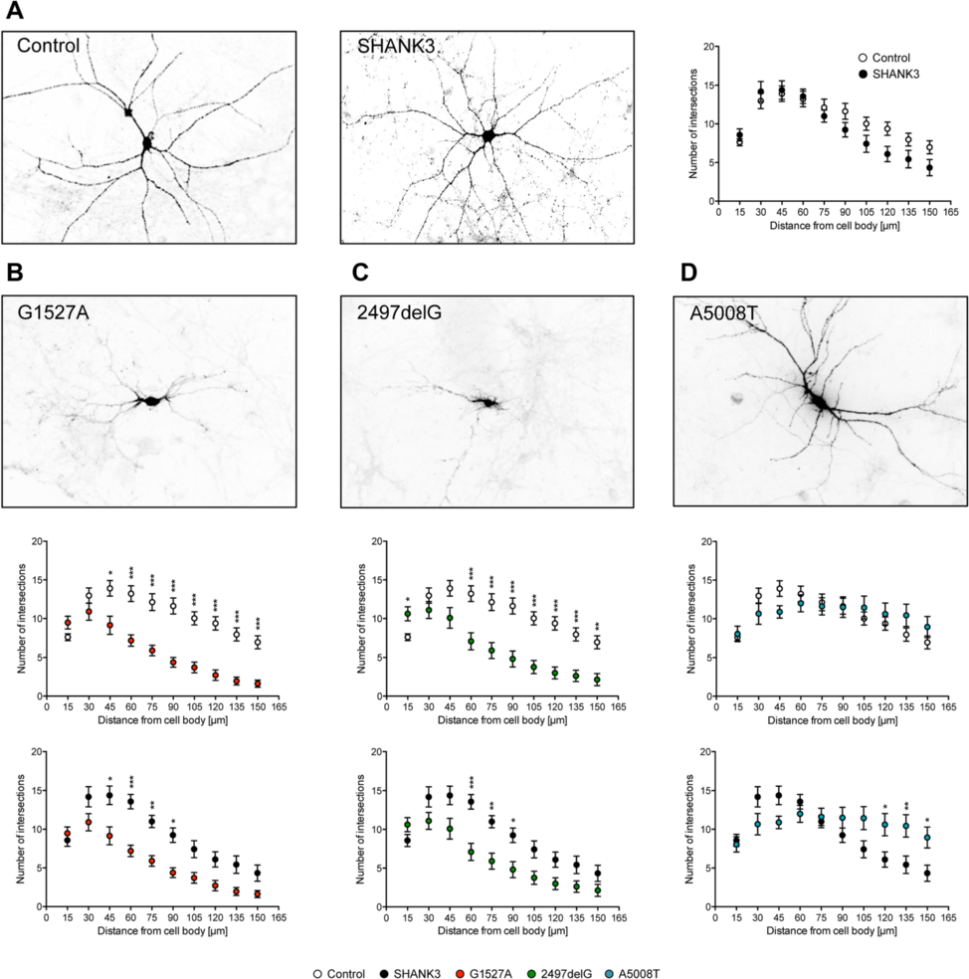
Overexpression of truncated SHANK3 variants results in distinct alterations of dendritic tree complexity. **(A-D)** Dendritic tree complexity of rat primary hippocampal neurons co-transfected with DenMark and either empty vector-based GFP (Control), full-length GFP-Myc-SHANK3 (SHANK3), or the truncated GFP-Myc-SHANK3 variants G1527A, 2497delG, and A5008T (DIV11-14). **(A)** Representative images of the DenMark signal in rat primary hippocampal neurons overexpressing either Control or SHANK3, as indicated. The panel on the right shows a Sholl analysis of Control (white-filled circles) *vs*. SHANK3 (black-filled circles). (B-D) Representative images of the DenMark signal in rat primary hippocampal neurons overexpressing either G1527A **(B)**, 2497delG **(C)**, or A5008T **(D)**, as indicated. The panels below each image show Sholl analyses either of Control (white-filled circles, upper panel) or SHANK3 (black-filled circles, lower panel) *vs*. the corresponding truncated SHANK3 variant (red-filled circles for G1527A in **(B)**, green-filled circles for 2497delG in **(C)**, and blue-filled circles for A5008T in **(D)**). *P < 0.05, **P < 0.01, ***P < 0.001 compared with either Control or SHANK3.

To assess neuronal morphology of transfected neurons, we first analyzed the dendritic tree. Primary dendrite number revealed no differences among all conditions (Additional file 6: Figure S4). Subsequent Sholl analysis showed that dendritic tree complexity was identical in Control and with overexpressed SHANK3 (Figure 3A). However, overexpression of either G1527A or 2497delG resulted in a much lower complexity of the dendritic tree when compared to either Control or SHANK3 (Figure 3B,C) indicating severely impaired dendritic branching. Interestingly, overexpression of A5008T did not show any gross alterations of dendritic arborization when compared to Control, but some enhanced branching of distal dendrites when compared to SHANK3 (Figure 3D).

### Overexpression of truncated SHANK3 variants results in distinct alterations of dendritic spines and synaptic contacts

**Figure 4:**
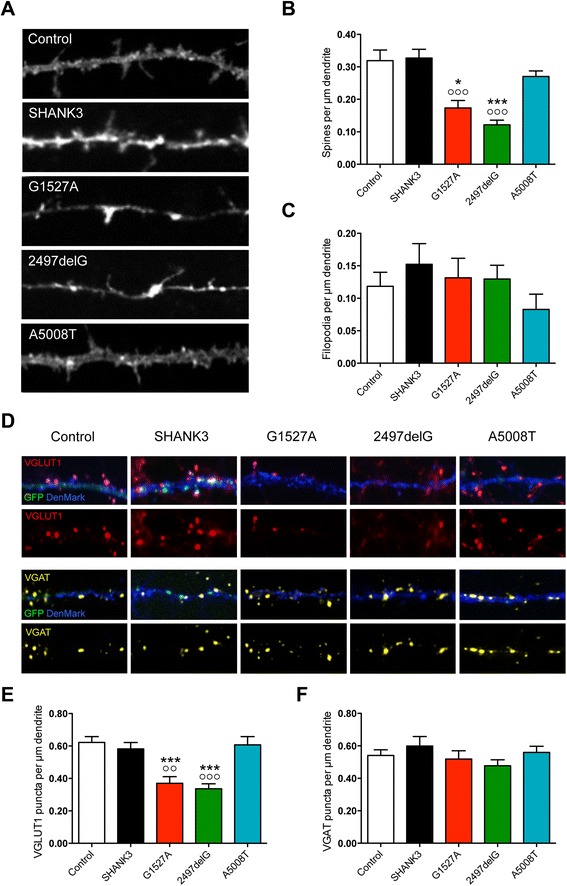
Overexpression of truncated SHANK3 variants results in distinct alterations of dendritic spines and synaptic contacts. Evaluation of dendritic spines **(A-C)** and synaptic contacts **(D-F)** in rat primary hippocampal neurons co-transfected with DenMark and either empty vector-based GFP (Control), full-length GFP-Myc-SHANK3 (SHANK3), or truncated GFP-Myc-SHANK3 variants (G1527A, 2497delG, A5008T) (DIV11-14). **(A)** Representative images of the DenMark signal in secondary dendrites of rat primary hippocampal neurons overexpressing either Control, SHANK3, G1527A, 2497delG, or A5008T as indicated. **(B)** Quantitative analysis of spine density. **(C)** Quantitative analysis of filopodia density. **(D)** Representative images of VGLUT1-positive (upper two rows) and VGAT-positive (lower two rows) presynaptic specializations among secondary dendrites of rat primary hippocampal neurons overexpressing either Control, SHANK3, G1527A, 2497delG, or A5008T, as indicated. **(E)** Quantitative analysis of VGLUT1-positive puncta density. **(F)** Quantitative analysis of VGAT-positive puncta density. White bars, Control; black bars, SHANK3; red bars, G1527A; green bars, 2497delG; and blue bars, A5008T. *P < 0.05 and ***P < 0.001 compared with Control; ^oo^ *P* < 0.01 and ^ooo^ *P* < 0.001 compared with SHANK3.

Further assessment of neuronal morphology included the evaluation of spines and filopodia among secondary dendrites of transfected neurons (Figure 4A,B,C). Overexpression of either G1527A or 2497delG resulted in a strong reduction in spine density when compared to either Control or SHANK3 (Figure 4B), while filopodia density remained unchanged (Figure 4C). In contrast, A5008T overexpression did not affect spine or filopodia density at all (Figure 4B,C). These data show that overexpression of either G1527A or 2497delG results in a specific loss of spines accompanied by a relative increase in the number of filopodia per μm dendrite (Figure 4B,C, for spine/filopodia and filopodia/spine ratios, see Table 1), while there was no significant effect of A5008T overexpression on spine or filopodia density (Figure 4B,C, for spine/filopodia and filopodia/spine ratios, see Table 1).

**Table 1:** **Spine/filopodia and filopodia/spine ratios of rat primary hippocampal neurons overexpressing different SHANK3 variants**

**Fusion protein**	**Spines/filopodia**	**Filopodia/spines**
Control	2.70	0.37
SHANK3	2.15	0.47
G1527A	1.32	0.76
2497delG	0.94	1.07
A5008T	3.26	0.31

We next analyzed presynaptic specializations among secondary dendrites of transfected neurons and discriminated excitatory and inhibitory contacts by immunostaining for either VGLUT1 or VGAT (Figure 4D,E,F). In line with our findings on spines, the dendritic contact sites for excitatory synapses, overexpression of either G1527A or 2497delG, resulted in a major loss of VGLUT1-positive puncta per μm dendrite, while VGAT puncta density remained unaffected (Figure 4D,E,F). In contrast, there was no detectable change in the density of either VGLUT1- or VGAT-positive puncta when A5008T was compared to either Control or SHANK3 (Figure 4D,E,F). These findings demonstrate that overexpression of both G1527A and 2497delG leads to a specific decrease in excitatory, but not inhibitory contacts, thus likely shifting the excitation/inhibition (E/I) balance towards inhibition. In contrast, no E/I imbalance phenotype can be implicated at this point when examining overexpression of A5008T in primary hippocampal culture.

## Discussion

In this study, we have described, for the first time, a novel truncating stop mutation in an individual with the diagnosis of both ASD and ID (c.5008A > T) (Table 2). In addition, *in vitro* analyses with this and two other recently identified truncating stop mutations (c.1527G > A and c.2497delG, [17]) addressed the question if distinct mutations in *SHANK3* result in mutation-specific or converging morphological phenotypes.

**Table 2:** **Genetic information on the three participants included into this study**

	**Sex**	**Diagnosis**	**Rearrangement**	**Aa change**	**Inheritance**	**Reference**
P1	M	ASD/ID	c.1527G > A	p.W509X	*De novo*	Soorya *et al*. 2013 [17]
P2	M	ID	c.2497delG	p.P834RfsX58	*De novo*	Soorya *et al*. 2013 [17]
P3	M	ASD/ID	c.5008A > T	p.K1670X	Not from mother	This study

Interestingly, we found that SHANK3a variants harboring either c.1527G > A or c.2497delG, thus lacking large but distinct parts of the protein’s C-terminus (Figure 1A), exhibit overlapping phenotypes in all morphological parameters investigated. When overexpressed, both truncated proteins exclusively accumulate in the nuclear compartment of transfected cells (Figures 2C,D, Additional file 5: Figure S3A,B) and this is accompanied by a severe reduction of dendritic tree complexity (Figure 3A,B,C). Moreover, we found a specific reduction of dendritic spine and excitatory, but not inhibitory synapse density (Figure 4A,B,C,D,E,F). These findings are in line with previous *in vitro* studies showing similar phenotypes for rodent homologue Shank3a variants harboring either c.3679_3680insG or c.3349C > T [23,25].

In contrast, none of these phenotypes was observed in neurons overexpressing SHANK3a haboring the c.5008A > T mutation. However, some minor alterations in neuronal morphology were detected, and interestingly, they were opposite from the ones related to c.1527G > A and c.2497delG as c.5008A > T produced slightly enhanced complexity of the distal dendritic tree (Figure 3D).

With respect to subcellular localization of truncated SHANK3, an important aspect to consider is domain composition. The SHANK3a c.5008A > T variant is only lacking a small part of the protein’s C-terminus, thereby exclusively disrupting the SAM domain (Figure 1A), a domain needed for the correct assembly of Shanks in the PSD [27]. In line with this, it still localizes to dendrites and excitatory synapses of transfected neurons in cluster-like structures, although with a much lower efficiency as compared to full-length SHANK3a (Figure 2E, Additional file 4: Figure S2A,B). The two other truncated SHANK3a variants analyzed here are lacking major parts of the C-terminus including distinct stretches of the proline-rich domain and - in all cases - the Homer binding site (a dendritic localization signal), a synaptic targeting element in between the Homer and cortactin binding sites and the SAM domain (Figure 1A). In line with this, each of these variants never forms cluster-like structures within dendrites or at spine synapses, but rather localizes to the nucleus of transfected neurons (Figure 2C,D).

A recent study on the transcriptional and functional complexity of the rodent *Shank3* gene convincingly delineates all rodent Shank3 isoforms (Shank3a-f) and reports distinct and/or overlapping phenotypes for each isoform with respect to subcellular distribution and neuronal morphology after overexpression in primary hippocampal neurons [37]. Summarizing what was observed, selected Shank3 isoforms either increase the number of spines and excitatory synapses or show the opposite effect, likely depending on their domain composition and subcellular localization. Intriguingly, the Shank3 isoform Shank3b, which only contains the ankyrin repeats, the SH3 domain, and the PDZ domain [37], resembles two of the shorter truncated SHANK3a variants described here. In primary hippocampal neurons, overexpressed Shank3b localizes to the nucleus accompanied by a reduced number of mature spines and excitatory synapses [37] - just as we report in this study for overexpression of SHANK3a harboring either c.1527G > A or c.2497delG mutations (Figures 2, 4) and as others have reported in previous studies for the rodent Shank3a homologue harboring either c.3679_3680insG or c.3349C > T [23,25].

Although the exact function of each isoform is yet to be determined, it can already be hypothesized at this point that in a healthy neuron, the expression of SHANK3 isoforms is fluid, adapting to the ever-changing needs of the cell. Hence, we can speculate that in neurons from individuals with heterozygous stop mutation in *SHANK3,* there will be disrupted functionality under specific conditions, due to expression of variant proteins at key stages.

Genotype-phenotype correlations can be complicated because of the potential for additional genetic variation contributing to more or less severe phenotypes. In fact, P1 has both an early truncating mutation in *SHANK3*, as well as a 17q duplication, and has one of the more severe presentations observed at the Seaver Autism Center. However, it is still of interest to note that the three individuals described here are all significantly affected, irrespective of the extent of the truncation. In fact, it is of particular interest that the A5008T variation is associated with a very mild cellular phenotype but with a severe behavioral phenotype in the participant. A better understanding of this association will require additional studies as noted below, although it is also possible that A5008T causes a different cellular phenotype, which has not been assessed in this study.

Transfection experiments as we have carried out here involve the modification of a single SHANK3 isoform (SHANK3a). In addition, we cannot assess other genetic variation that may alter severity and we cannot exclude the possibility that in patient cells, both c.1527G > A and c.2497delG - as being located in coding exons upstream the natural stop codon - lead to SHANK3 haploinsufficiency due to degradation of the truncated proteins by the nonsense-mediated mRNA decay machinery [39]. It will therefore be essential to differentiate neurons from induced pluripotent stem cells (iPSCs) of participants affected by any of the aforementioned stop mutations and analyze neuronal morphology as well as domain composition, subcellular localization, and mRNA and protein expression of endogenous SHANK3. However, neuronal culture will never completely reflect the impact of a given mutation on the network level *in vivo*. Hence, another essential approach to study mutations in *SHANK3* would be to generate animal models that carry the human mutation. With respect to stop mutations disrupting exon 21 of *SHANK3,* such as c.2497delG from this study, the recently published *Shank3*^*ΔC/ΔC*^ mouse, which is an exon 21 deletion model [40], may already provide some insights. Importantly, increases in C-terminally truncated Shank3 variants are detectable in hippocampal lysates from these animals. Compared to other *Shank* mutant mice [41,42], only minimal social deficits were observed in these animals, and increased repetitive behavior was only evident at a certain age (10 to 13 months). CA1 pyramidal neurons did not exhibit a morphological phenotype. The *Shank3*^Δ*C*/Δ*C*^ mutants did clearly show impaired spatial learning and corresponding abnormalities in hippocampal CA3-CA1 physiology though, including decreased long-term potentiation (LTP) and decreased NDMA receptor-mediated synaptic transmission. However, it has to be noted that only analyses of homozygous mutants have been reported so far. Studying heterozygous mutants - as the more ‘human-like’ model - would possibly reveal different phenotypes not only in this but also in other models mimicking human mutations in *SHANK3*.

## Conclusions

Our *in vitro* overexpression data show that the location of a stop mutation within the *SHANK3a* sequence determines both subcellular localization of the truncated protein and the morphological phenotype of the transfected neuron.

Considering domain composition of SHANK3, our data support previous studies [15,23-29,37] and strengthen the fact that only SHANK3 variants with an intact C-terminus including the Homer binding site, a synaptic targeting element in between the Homer and cortactin binding sites and the SAM domain, correctly and efficiently localize to synapses. Any disruption of these domains results in a distinct phenotype of dendritic and synaptic morphology. Interestingly, our data imply that loss of the Homer and cortactin binding sites is sufficient to induce nuclear accumulation of the corresponding SHANK3 variants. This again supports previous studies proposing that SHANK3 might not only serve as a synaptic but also serve as a nuclear protein [25,37] - although its exact nuclear function still remains an enigma.

Considering recent findings on the complexity of both subcellular localization and distinct morphological impact of different rodent Shank3 isoforms in healthy neurons [37], we propose that any heterozygous deleterious mutation in *SHANK3* will lead to altered SHANK3 isoform expression and thereby result in distinct spatial and temporal perturbations of SHANK3-dependent cellular processes. Although we cannot further investigate such a proposed patho-mechanistic model in the *in vitro* assay used in this study, its effect on the network level within the complex and dynamic architecture of the brain would be predicted to be substantial. Moreover, it would also be consistent with our observation that the clinical phenotype remains quite severe in all three individuals included in this study, with clear overlap in the symptom presentation, regardless of whether most or only part of *SHANK3* is missing.

## Additional files

Additional file 1: Table S1. Descriptive and diagnostic data by participant: Mullen Scales of Early Learning, Nonverbal IQ estimate, Vineland Adaptive Behavior Scales, ADI-R, and ADOS-2. Additional file 2: Table S2. Medical features (a) and dysmorphic features (b) of the three participants. Additional file 3: Figure S1. Additional biochemical expression analysis of SHANK3 variants in HEK293T cells. The anti-GFP antibody detects GFP at the right size after overexpression of the empty vector in HEK293T cells (A). Only the fusion proteins SHANK3, 2497delG and A5008T, but not G1527A, can be biochemically detected by the self-made anti-Shank3 PRC antibody (B). Successful detection of G1527A (as well as rat Shank3a, SHANK3, and 2497delG) was accomplished with a novel self-made anti-Shank3 N-term antibody (C). kDa, kilodalton. Additional file 4: Figure S2. Additional subcellular distribution analysis of SHANK3 variants in rat primary hippocampal neurons. Quantitative analysis of GFP cluster density (A) and the density of GFP clusters overlapping with VGLUT1 signals (B) among secondary dendrites of rat primary hippocampal neurons filled with DenMark and overexpressing either SHANK3 (black bars) or A5008A > T (blue bars) as indicated (DIV11-14). ****P* < 0.001 compared with SHANK3. Additional file 5: Figure S3. Nuclear accumulation of the SHANK3 variants G1527A and 2497delG in HEK293T cells. (A, B) Subcellular distribution of SHANK3, G1527A, 2497delG and A5008T in HEK293T cells. (A) Merged pictures of the GFP signal, the phalloidin signal (visualizing the actin cytoskeleton) and the DAPI signal (upper row), the GFP signal alone (middle row) and the DAPI signal alone (lower row). Note strong overlap of both G1527A and 2497delG with the DAPI signal. (B) Biochemical detection of overexpressed G1527A and 2497delG in cytosolic (Cyt) and nuclear (Nuc) fractions isolated from HEK293T cells using an anti-GFP antibody. Anti-GAPDH and anti-Histone H3 antibodies were used to control fractionation as indicated. kDa, kilodalton. Additional file 6: Figure S4. Primary dendrite number of rat primary hippocampal neurons overexpressing SHANK3 variants. Quantitative analysis of the number of primary dendrites of rat primary hippocampal neurons filled with DenMark and overexpressing either Control (white bar), SHANK3 (black bar), G1527A (red bar), 2497delG (green bar), or A5008A > T (blue bar) as indicated (DIV11-14). No statistical differences were observed.

## Abbreviations

ADI-R: Autism Diagnostic Interview Revised
ADOS: Autism Diagnostic Observation Schedule
Ank: ankyrin repeats
ASD: autism spectrum disorder
cDNA: complementary DNA
DSM: Diagnostic and Statistical Manual for Mental Disorders
E18: embryonic stage 18
EEG: electroencephalography
GERD: gastroesophageal reflux disease
H + C: Homer and cortactin binding sites
ID: intellectual disability
iPSCs: induced pluripotent stem cells
IRB: Institutional Review Board
LTP: long-term potentiation
MRI: magnetic resonance imaging
N-term: N-terminus
P1: participant 1
P2: participant 2
P3: participant 3
PDZ: PSD-95/Dlg-1/ZO-1
PECS: Picture Exchange Communication System
PFA: paraformaldehylde
PRC: proline-rich clusters
Pro: proline-rich region
PSD: postsynaptic density
SAM: sterile alpha motif
SCZ: schizophrenia
SH3: Src-homology 3
