# Supplementary material for: Phenotypic and functional analysis of SHANK3 stop mutations identified in individuals with ASD and/or ID
Source: Mol Autism. 2015 Apr 29;6:23. doi: 10.1186/s13229-015-0020-5 (PMC4455919; doi:10.1186/s13229-015-0020-5)
Supplement: Additional file 1: Table S1. — Descriptive and diagnostic data by participant: Mullen Scales of Early Learning, Nonverbal IQ estimate, Vineland Adaptive Behavior Scales, ADI-R, and ADOS-2. [file 13229_2015_20_MOESM1_ESM.pdf]

Additional file 1: Table S1. Descriptive and diagnostic data by patient: Mullen Scales of Early Learning, Nonverbal IQ estimate, Vineland Adaptive Behavior Scales, ADI-R, and ADOS-2.

Participant	Age (yrs: mo)	Mullen Scales of Early Learning						Vineland Adaptive Behavior Scales-II (Standard Score)					ADI-R[&]			ADOS-2 Module 1				Diagnosis	
		Gross Motor	Visual Reception	Fine Motor	Receptive Lang.	Expressive Lang.	NVIQ	ABC	Communication	Socialization	Daily Living	Motor	A: Social	B: Comm	C: RRBIs	SA Total	RRB Total	Overall (SA+ RRB) Total	Severity Score	DSM-IV	Consensus
P1 (G1527A)	5:1	n/a	12	15	8	6	19.67	45	42	49	38	59	23	13	6	19	5	24	8	2	2
P2 (2497delG)	7:1	20	14	18	13	5	16.51	49	45	51	52	40	17	10	7	2	3	5	2	0	0
P3 (A5008T)	12:2	30	20	20	8	9	13.63	44	42	42	50	64	29	13	4	18	2	20	7	2	2

[&]ADI-R cutoff scores for autism are: Social = 10, Communication (nonverbal) = 7, Repetitive behaviors and restricted interests = 3
[&]ADOS-2 Module 1 cutoff scores on Overall Total Score for autism and ASD are 16 and 11, respectively.

Abbreviations: ABC= Adaptive Behavior Composite (Vineland); ADI-R=Autism Diagnostic Interview-Revised; A: Social= Qualitative Abnormalities in Reciprocal Social Interaction; B: Comm= Qualitative Abnormalities in Communication; C: RRBIs= Restricted, Repetitive, and Stereotyped Patterns of Behavior; ADOS= Autism Diagnostic Observation Schedule; SA Total= Social Affect Total; RRB Total=Restricted and Repetitive Behavior Total; Consensus=Consensus diagnosis; DSM-IV= Diagnostic and Statistical Manual of Mental Disorders-IV; IQ= intellectual quotient; NVIQ= Nonverbal IQ; n/a= not available; yrs=years.
