# Supplementary material for: Phenotypic and functional analysis of SHANK3 stop mutations identified in individuals with ASD and/or ID
Source: Mol Autism. 2015 Apr 29;6:23. doi: 10.1186/s13229-015-0020-5 (PMC4455919; doi:10.1186/s13229-015-0020-5)
Supplement: Additional file 2: Table S2. — Medical features (a) and dysmorphic features (b) of the three participants. [file 13229_2015_20_MOESM2_ESM.pdf]

Additional file 2: Table S2

Medical Features

Participant	Mutation	Seizures	Hypotonia	Recurring infection	Constipation/ diarrhea	Gastroesophageal reflux disease	History or regression	Gait abnormalities	Brain MRI abnormalities	Sleep disturbance
1	G1527A		x					x	x	
2	2497delG	x	x	x	x	x	x	x	x	x
3	A5008T	x	x	x	x	x	x	x	x	x

Dysmorphic features

Participant	Mutation	Long eyelashes	Ear anomalies	Full lips	Epicanthal folds	Macrocephaly	Dolicocephaly	Wide nasal bridge	Hypertelorism	Short stature	2-3 Syndactyly of toes	5th Finger clinodactyly	Deep set eyes	Broad nasal bridge
1	G1527A	x	x	x	x	x		x	x					x
2	2497delG						x			x				
3	A5008T										x	x	x	
