# Supplementary figures and images for: Phenotypic and functional analysis of SHANK3 stop mutations identified in individuals with ASD and/or ID
Source: Mol Autism. 2015 Apr 29;6:23. doi: 10.1186/s13229-015-0020-5 (PMC4455919; doi:10.1186/s13229-015-0020-5)

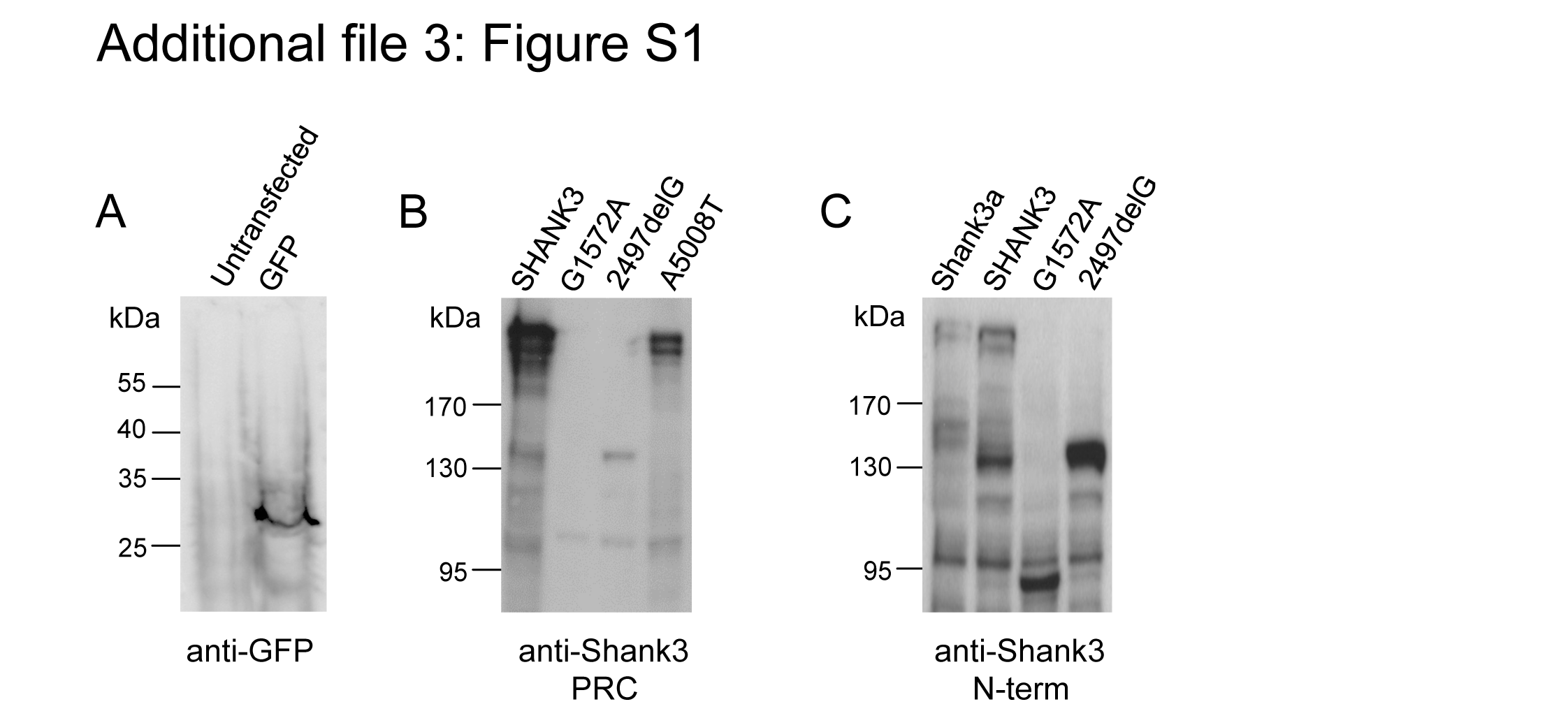

Supplement: Additional file 3: Figure S1. — Additional biochemical expression analysis of SHANK3 variants in HEK293T cells. The anti-GFP antibody detects GFP at the right size after overexpression of the empty vector in HEK293T cells (A). Only the fusion proteins SHANK3, 2497delG and A5008T, but not G1527A, can be biochemically detected by the self-made anti-Shank3 PRC antibody (B). Successful detection of G1527A (as well as rat Shank3a, SHANK3, and 2497delG) was accomplished with a novel self-made anti-Shank3 N-term antibody (C). kDa, kilodalton. [file 13229_2015_20_MOESM3_ESM.png]

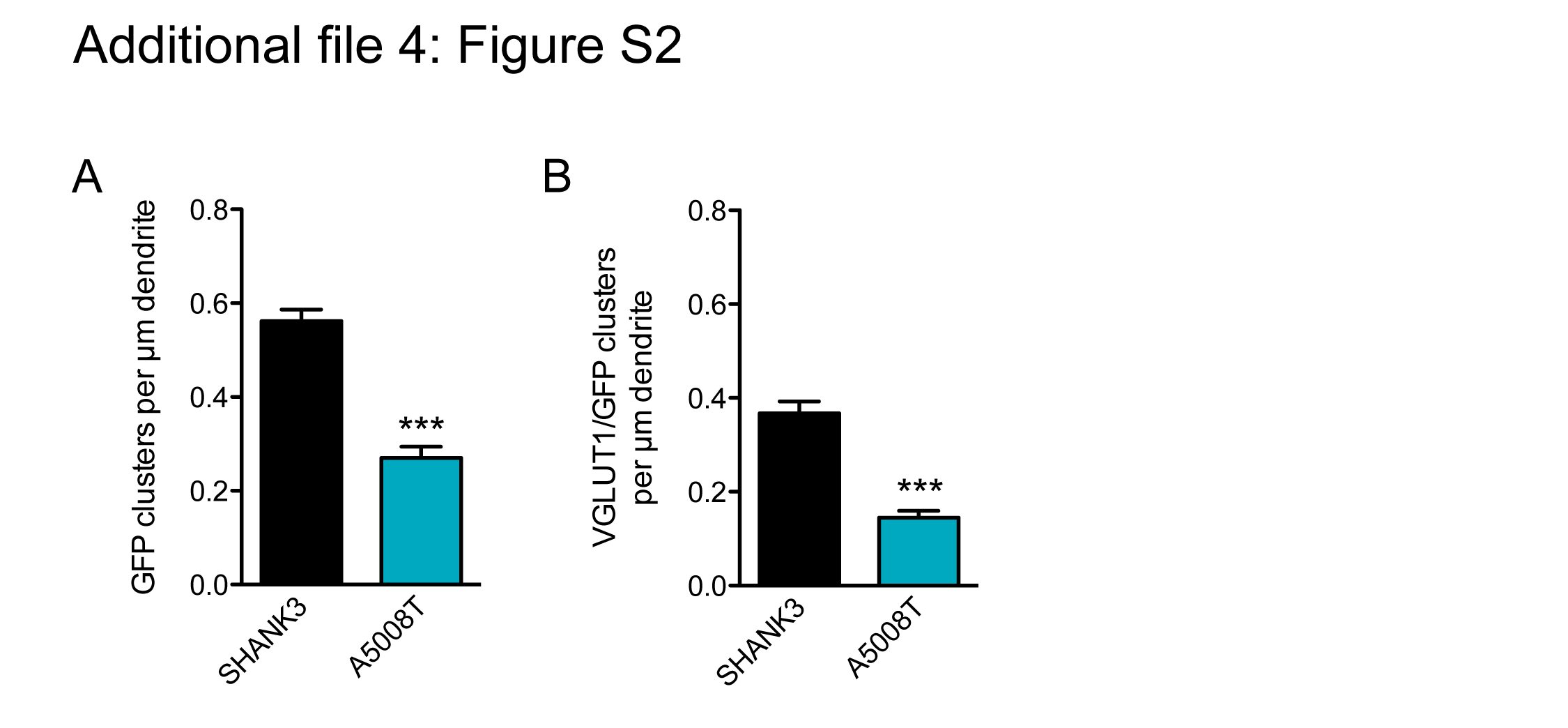

Supplement: Additional file 4: Figure S2. — Additional subcellular distribution analysis of SHANK3 variants in rat primary hippocampal neurons. Quantitative analysis of GFP cluster density (A) and the density of GFP clusters overlapping with VGLUT1 signals (B) among secondary dendrites of rat primary hippocampal neurons filled with DenMark and overexpressing either SHANK3 (black bars) or A5008A > T (blue bars) as indicated (DIV11-14). ***P < 0.001 compared with SHANK3. [file 13229_2015_20_MOESM4_ESM.png]

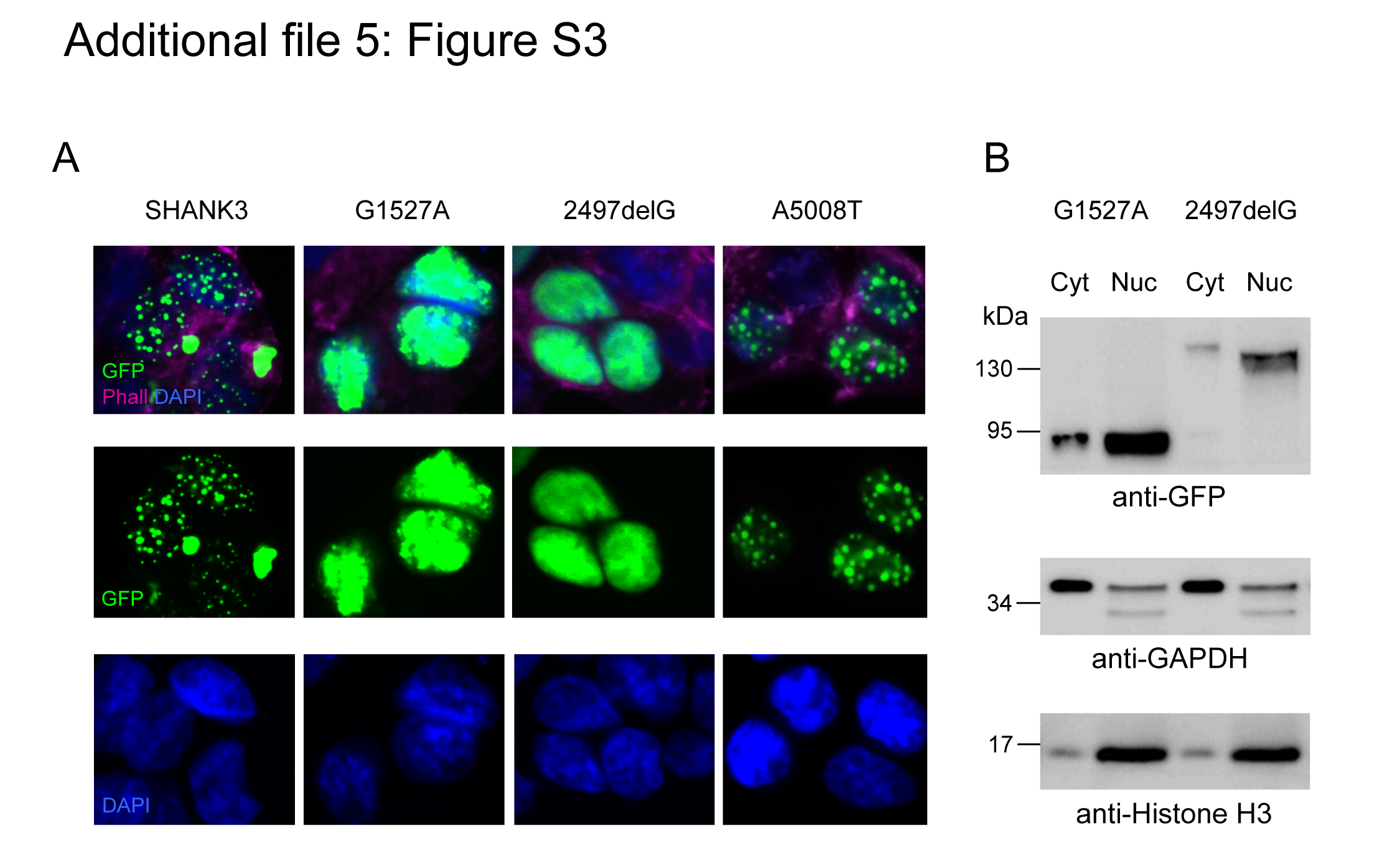

Supplement: Additional file 5: Figure S3. — Nuclear accumulation of the SHANK3 variants G1527A and 2497delG in HEK293T cells. (A, B) Subcellular distribution of SHANK3, G1527A, 2497delG and A5008T in HEK293T cells. (A) Merged pictures of the GFP signal, the phalloidin signal (visualizing the actin cytoskeleton) and the DAPI signal (upper row), the GFP signal alone (middle row) and the DAPI signal alone (lower row). Note strong overlap of both G1527A and 2497delG with the DAPI signal. (B) Biochemical detection of overexpressed G1527A and 2497delG in cytosolic (Cyt) and nuclear (Nuc) fractions isolated from HEK293T cells using an anti-GFP antibody. Anti-GAPDH and anti-Histone H3 antibodies were used to control fractionation as indicated. kDa, kilodalton. [file 13229_2015_20_MOESM5_ESM.png]

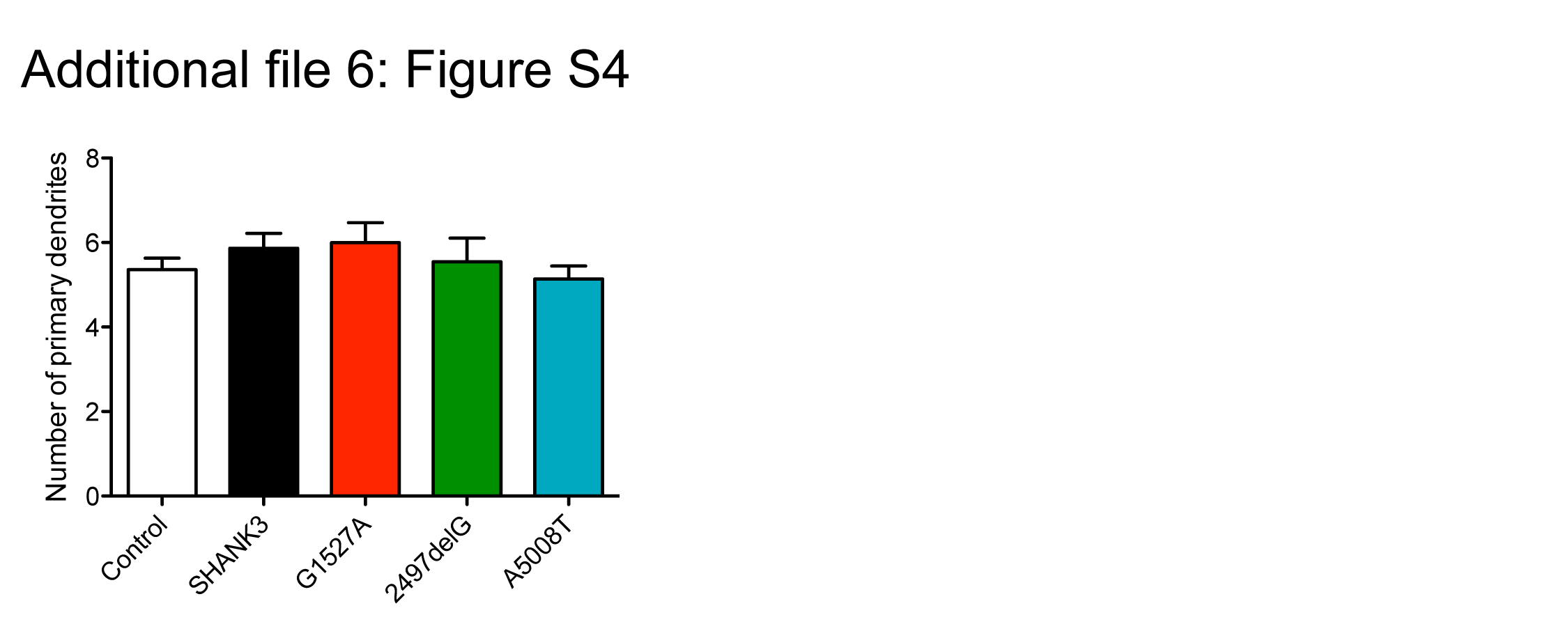

Supplement: Additional file 6: Figure S4. — Primary dendrite number of rat primary hippocampal neurons overexpressing SHANK3 variants. Quantitative analysis of the number of primary dendrites of rat primary hippocampal neurons filled with DenMark and overexpressing either Control (white bar), SHANK3 (black bar), G1527A (red bar), 2497delG (green bar), or A5008A > T (blue bar) as indicated (DIV11-14). No statistical differences were observed. [file 13229_2015_20_MOESM6_ESM.png]
